# Maternal depression during pregnancy and cord blood DNA methylation: findings from the Avon Longitudinal Study of Parents and Children

**DOI:** 10.1038/s41398-018-0286-4

**Published:** 2018-11-07

**Authors:** A. C. Viuff, G. C. Sharp, D. Rai, T. B. Henriksen, L. H. Pedersen, K. J. Kyng, N. H. Staunstrup, A. Cortes, A. Neumann, J. F. Felix, H. Tiemeier, V. W. V. Jaddoe, C. L. Relton

**Affiliations:** 1 Perinatal Epidemiology Research Unit, Department of Clinical Medicine, Health, Aarhus University, Aarhus, Denmark; 2 MRC Integrative Epidemiology Unit, University of Bristol, Bristol, UK; 3 School of Oral and Dental Sciences, University of Bristol, Bristol, UK; 4 Population Health Sciences, Bristol Medical School, University of Bristol, Bristol, UK; 5 Avon & Wiltshire Partnership NHS Mental Health Trust, Bristol, UK; 6 Department of Paediatrics, Aarhus University Hospital, Aarhus, Denmark; 7 Department of Obstetrics and Gynaecology, Aarhus University Hospital, Aarhus, Denmark; 8 The Lundbeck Foundation Initiative for Integrative Psychiatric Research, iPSYCH, Aarhus, Denmark; 9 Translational Neuropsychiatric Unit, Aarhus University Hospital, Risskov, Denmark; 10 Department of Child and Adolescent Psychiatry and Psychology, Erasmus MC, Rotterdam, The Netherlands; 11 Department of Epidemiology, Erasmus MC, Rotterdam, The Netherlands; 12 The Generation R Study Group, Erasmus MC, Rotterdam, The Netherlands; 13 Department of Pediatrics, Erasmus MC, Rotterdam, The Netherlands

## Abstract

Up to 13% of women may experience symptoms of depression during pregnancy or in the postpartum period. Depression during pregnancy has been associated with an increased risk of adverse neurodevelopmental outcomes in the child and epigenetic mechanisms could be one of the biological pathways to explain this association. In 844 mother–child pairs from the Avon Longitudinal Study of Parents and Children, we carried out an epigenome-wide association study (EWAS) to investigate associations between prospectively collected data on maternal depression ascertained by the Edinburgh Postnatal Depression Scale in pregnancy and DNA methylation in the cord blood of newborn offspring. In individual site analysis, we identified two CpG sites associated with maternal depression in the middle part of pregnancy. In our regional analysis, we identified 39 differentially methylated regions (DMRs). Seven DMRs were associated with depression at any time point during pregnancy, 7 associated with depression in mid-pregnancy, 23 were associated with depression in late pregnancy, and 2 DMRs were associated with depression throughout pregnancy. Several of these map to genes associated with psychiatric disease and brain development. We attempted replication in The Generation R Study and could not replicate our results. Although our findings in ALSPAC suggest that maternal depression could be associated with cord blood DNA methylation the results should be viewed as preliminary and hypothesis generating until further replicated in a larger sample.

## Introduction

Up to 13% of women experience symptoms of depression during pregnancy or postpartum^1,2^. Depression during pregnancy has been associated with an increased risk of adverse neurodevelopmental outcomes in the offspring^3–5^. Children of mothers with depression during pregnancy have also been shown to be more likely to develop a psychiatric disorder later in life^6–8^. The biological mechanisms behind the association between prenatal maternal depression and predisposition to later behavioural problems, learning difficulties, and psychiatric illness in the offspring have been extensively discussed, and it has been hypothesised, that epigenetic pathways could be one of the biological pathways involved^9^. Epigenetics is the study of potentially heritable molecular modifications to DNA and histone proteins that can affect gene expression without change to the underlying DNA sequence^10^. The most frequently studied epigenetic phenomenon is DNA methylation where CpG dinucleotides undergo a process of cytosine methylation which can alter chromatin accessibility and thereby gene transcription^10^.

Timing of exposure to maternal depression may be important in understanding the causal relationship. Human studies suggest that the second and early third trimesters are the more sensitive periods when exposures to stressful life events increase the risk of offspring illness^11^, although the evidence is inconsistent^12^. Studies investigating candidate genes (*NR3C1 and SLC6A4*) in relation to maternal depression during pregnancy have suggested trimester-specific differences in methylation patterns in the cord blood of the offspring^13,14^.

The aim of this study was to test the hypothesis that prenatal maternal depression would be associated with epigenome-wide differences in methylation in the cord blood of newborns in a large birth cohort study.

## Methods

### Participants

The Avon Longitudinal Study of Parents and Children (ALSPAC) recruited 14,541 pregnant women resident in Avon, UK with an expected delivery date falling between 1 April 1991 and 31 December 1992 (ref. ^15^). During pregnancy the included women were sent up to four self-completion postal questionnaires. At least one questionnaire was completed by 14,119 women and 11,545 women completed all four questionnaires^16^. These questionnaires included questions on lifestyle, mental health, diet, medication use, education, occupation, ethnicity, alcohol, and tobacco use. Two of the questionnaires administered around the 18th and the 32nd week of pregnancy contained questions concerning depression. The study website contains details of all data that is available through a fully searchable data dictionary^17^ (http://www.bristol.ac.uk/alspac/researchers/). Ethical approval for the study was obtained from the ALSPAC Ethics and Law Committee and the Local Research Ethics Committees.

**Fig. 1:**
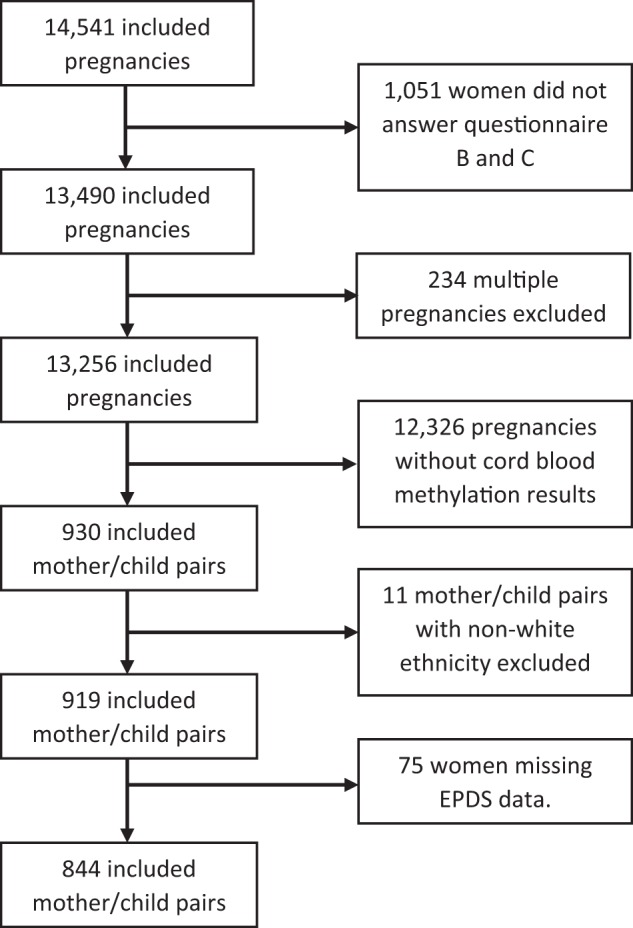
**Flowchart illustrating the selection of participants from ALSPAC**

We included women with singleton pregnancies where the woman had completed both questionnaires containing questions on depression during pregnancy and had a child with cord blood DNA methylation data. ALSPAC includes very few pregnancies of non-Caucasian ethnicity, so we excluded those from the study (Fig. 1).

### Measure of maternal depression

The postal questionnaires at 18 and 32 weeks of pregnancy included the 10-item Edinburgh Postnatal Depression Score (EPDS)^18^. The EPDS is a validated questionnaire to screen women for depression postpartum as well as during pregnancy^19^. Scores of ≥12 have a high correlation to clinically diagnosed depressive disorders^20^. A total of 844 women had data on EPDS scores and offspring cord blood DNA methylation (Fig. 1).

In our primary analysis we investigated whether screening positive for depression (EPDS ≥ 12) at either time point during pregnancy was associated with differential cord blood DNA methylation. Therefore, offspring of women who had an EPDS ≥ 12 at either week 18 or week 32 were considered exposed and these women will be referred to as depressed throughout this paper. We did three secondary analyses investigating the methylation differences in children of mothers with depression in mid-pregnancy only (EPDS ≥ 12 at week 18 and ≤12 at week 32), late in pregnancy only (EPDS ≥ 2 at week 32 and ≤12 at week 18), and at both time points in pregnancy (EPDS ≥ 12 at both weeks 18 and 32). The reference was offspring of women with EPDS < 12 at both week 18 and week 32.

### Methylation data

The procedure for collecting and storing cord blood samples is described elsewhere^21^. The DNA methylation analysis and the preprocessing of data were performed at the University of Bristol as a part of the ARIES project^22^. The ARIES project holds DNA methylation data on just under 1000 cord blood samples. DNA methylation was measured in cord blood samples using the Illumina Infinium HumanMethylation 450K BeadChip assay^23^. This platform analyses the methylation level of over 485,000 CpG sites in one array using bisulfite converted genomic DNA. The level of methylation is reported as a beta-value (*β*-value) ranging from 0 (completely unmethylated) to 1 (completely methylated).

Data were pre-processed in R (version 3.0.1) using the WateRmelon package^24^ and functional normalisation was performed using the Meffil package in R^25^ as an attempt to reduce non-biological differences between probes. We removed 11,607 probes mapping to the Y or X chromosomes or because they were SNPs included on the array for control purposes. In all, 2626 probes with a detection *p*-value >0.05 were also removed. We identified extreme outliers in the methylation data using the Tukey method^26^. This method uses the interquartile range (IQR) and extreme outliers are defined as values outside first quartile−3×IQR and third quartile+3×IQR. These probes were set as missing in the dataset.

### Covariates

We adjusted for potential confounders, which we selected with the help of Directed Acyclic Graphs (DAGs)^27^. The potential confounders were maternal education, parity, smoking, maternal pre-pregnancy BMI, and maternal age at delivery. We also adjusted for estimated cell proportions in the cord blood samples and technical batch. Maternal education was defined as having achieved below A-levels or A-levels and above. Parity was defined as no children or ≥1 child. Smoking was defined as any smoking during pregnancy or no smoking during pregnancy. Maternal age was included as a continuous variable. Cell proportions were estimated using the method developed by Houseman et al.^28^ and the cord blood reference panel published by Bakulski et al.^29^. We adjusted for technical batch using surrogate variable analysis (SVA)^30^. Using the *sva* package in R we estimated 10 surrogate variables which are covariates constructed from the data to adjust for unknown and un-modelled factors associated with the technical batch. Those surrogate variables not associated with the trait of interest are included as covariates in the regression analysis.

### Statistical analysis

#### Single site regression analyses

Linear regression was applied to investigate the association between maternal depression and methylation changes in the cord blood of the newborn. These EWASs were conducted in R version 3.0.2.

In our main analysis, DNA methylation (untransformed *β*-values) was the outcome. We ran four models to interrogate associations between depression at any time during pregnancy and DNA methylation: (1) adjusted for batch; (2) adjusted for batch and estimated cell proportion types; (3) adjusted for batch and covariates: maternal age, maternal pre-pregnancy BMI, parity, smoking, and maternal education; (4) adjusted for batch, covariates, and cell proportion types.

In secondary analyses, we applied the same four models, defining our exposure as depression in mid-pregnancy only, late pregnancy only, and depression at both time points.

*p*-Values were adjusted for genome-wide significance using false discovery rate (FDR) adjustment and only sites with FDR-corrected *p*-values <0.05 were considered statistically significant.

#### Regional analyses

When multiple adjacent CpG sites are differentially methylated, it is referred to as a differentially methylated region (DMR)^31^. DMRs may be more biologically important than differential methylation of individual, isolated CpGs, and region-based approaches are statistically more powerful with a lower rate of false positive findings compared to EWAS at individual CpGs. Therefore, in addition to the single-site analyses, we also performed regional analyses using Comb-P^32^. This method combines adjacent *p*-values from the single CpG analysis, corrects for correlation with neighbouring CpGs within 1000 bp applying the Stouffer-Liptak method, and adjusts for multiple testing using a Sidak correction^33^.

For each exposure of interest (depression at any time, mid-pregnancy, late pregnancy, and at both time points), we performed regional analyses on the results of the single-site analyses obtained using the fully adjusted model (model 4).

#### Enrichment analysis

We used the missMethyl R package^34^ to perform gene set enrichment analysis within the DMRs associated with depression in each of the four exposures of interest. This considers the differing number of sites associated with each gene on the Illumina 450K array. We tested for enrichment of any Gene Ontology (GO)^35^ classification term and Kyoto Encyclopaedia of Genes and Genomes (KEGG) pathways^36^. *p*-Values were adjusted for multiple testing using the FDR method. Only terms with FDR-corrected *p*-values <0.05 were considered significant.

#### Replication

We used data from the Generation R Study^37^ based in Rotterdam, the Netherlands for replication of our initial observations in ALSPAC. The Generation R Study is a population-based prospective cohort study from foetal life onwards. It includes 9778 women and their children, born between April 2002 and January 2006. The study has been approved by Medical Ethical Committee of Erasmus MC, University Medical Center Rotterdam, and written consent was obtained for all participants.

The Generation R Study Biobank has DNA methylation results from 1339 cord blood samples measured using the Illumina 450K Infinium BeadChip^38^. The depression subscale of the Brief Symptom Inventory (BSI)^39^ was completed at 20 weeks of pregnancy. This timing corresponds with the EPDS score from week 18 in ALSPAC. The BSI is a 53-item version of the Symptom Checklist-90 (SCL-90) that measures emotional-behavioural functioning. Information about maternal education, parity, and smoking during pregnancy was collected by self-reported questionnaires during pregnancy. Maternal education was classified in two levels: A-level or above (post-age 16 or above) and below A-level (education level of age 16). Parity was defined as no children or ≥1 child, and smoking during pregnancy as no smoking in pregnancy or any smoking during pregnancy. Pre-pregnancy BMI was calculated based on maternal pre-pregnancy weight, obtained by self-reported questionnaires, and maternal height measured at enrolment. Maternal age at delivery was calculated based on the date of birth of the mother and the child (Table S1).

A single-site regression analysis of the Generation R study methylation data was performed as well as a regional analysis as described above using the fully adjusted model. Cell proportions were adjusted for using the Houseman method with the reference panel for cord blood published by Bakulski et al.^29^. Plate ID (*n* = 27) was included in models as a covariate to adjust for technical batch. We used these results to do a look up of the results from our single site analysis and our DMR analysis in ALSPAC.

#### Methylation quantative trait loci analysis

When performing EWAS studies it must be considered that the methylation variation may partly be explained by underlying genetic variation. DNA sequences that are associated with patterns in DNA methylation are referred to as methylation quantative trait loci (mQTLs)^40^. These can act either in *cis* (within 1 MB either side of the CpG probe) or *trans* (further than 1MB from the CpG probe).

We looked up any maternal depression-associated CpG sites in a database of mQTLs (http://www.mqtldb.org/)^41^ previously identified in the cord blood of 771 ALSPAC children^41^. mQTLs were included in the database if the association between the SNP and the CpG had a *p*-value <1 × 10^–7^.

## Results

We had complete data on exposure to maternal depression during pregnancy and methylation changes in the cord blood of the offspring in 844 mother–child pairs in ALSPAC. Of these women 204 screened positive for depression at any time point in pregnancy: 53 women were depressed only in mid-pregnancy, 85 women only in late pregnancy, and 66 women at both time points. Our reference group consisted of 644 women classified as not depressed at any point during pregnancy.

More women with depression reported smoking during pregnancy than women in the reference group. Women in the reference group had a higher level of education than women with depression. The groups were comparable regarding other characteristics (Table 1).

**Table 1:** Characteristics of the included women

*n* = 844	Depression any time in pregnancy (*n* = 204) (EPDS ≥ 12)	Depression in mid- pregnancy^a^ (*n* = 53)	Depression in late pregnancy^b^ (*n* = 85)	Depression throughout pregnancy^c^ (*n* = 66)	No depression in pregnancy (*n* = 644) (EPDS < 12)
Age (years) (mean (95% CI))	29.4(28.7; 30)	29.4(28.1; 30.7)	29.7(28.6; 30.8)	28.8(27.6; 30.1)	29.7 (29.4; 30.1)
BMI (mean ((95% CI))	22.7(22.2; 23.2)	23.2(22.2; 24.2)	22.1(21.4; 22.8)	23.1(22; 24.2)	22.9(22.6; 23.2)
**Parity (** ***n*** **)**					
No children	82 (41%)	23(44%)	29(35%)	30(45%)	314(49%)
≥1 child	116 (56%)	28(53%)	53(62%)	34(52%)	312(48%)
**Smoking in pregnancy (** ***n*** **)**					
No smoking	150 (74%)	39 (74%)	68 (80%)	43 (65%)	584 (91%)
Any smoking	52 (25%)	14 (26%)	15 (18%)	23 (35%)	60 (9%)
**Education Level**					
O-levels/GCSE	117 (57%)	26 (49%)	47 (55%)	44 (67%)	312 (49%)
A-levels and above	85 (42%)	25 (47%)	38 (45%)	22 (33%)	331 (51%)

^a^EPDS ≥ 12 at 18 weeks only ^b^ EPDS ≥ 12 at 32 weeks only ^c^ EPDS ≥ 12 at 18 and 32 weeks

### Single site regression analyses

In our main analysis (depression at any time point in pregnancy), we found no significantly differentially methylated single CpG sites (FDR-corrected *p*-value <0.05) in any of the four models.

**Fig. 2:**
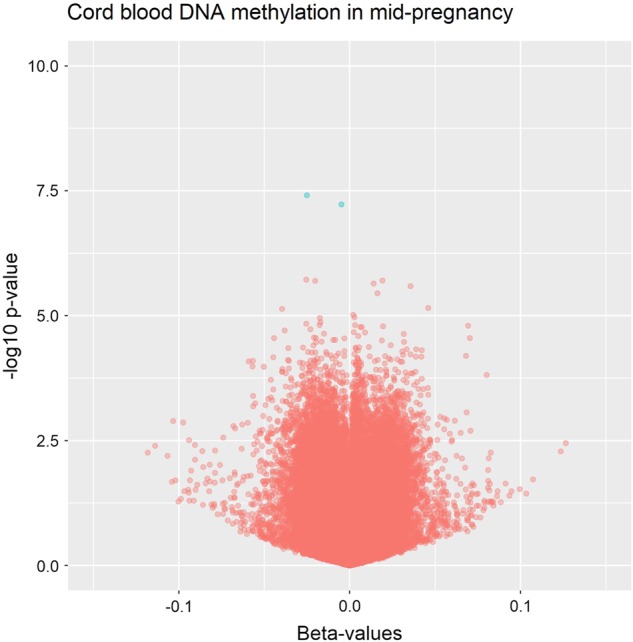
**Volcano plot illustrating the two significant single CpG sites from the linear regression analysis specific to depression in mid-pregnancy**

When investigating depression in mid-pregnancy (up to week 18), we identified two CpGs with FDR-corrected *p*-values <0.05 (Fig.2). Methylation of cg08667740, located on a CpG shore in the body of the *CYBA* gene, was 2.5% (*β* = −0.025; *p* = 3.9 × 10^−8^) lower in the umbilical cord blood of children exposed to maternal depression in mid-pregnancy, relative to unexposed children. Methylation of cg22868225, in the body of the *PRKCZ* gene, was 0.5% lower (*β* = −0.005; *p* = 5.89 × 10^−8^). This association was present in all four models. There were no CpGs with FDR-corrected *p*-value <0.05 in the single-site EWAS of depression in late pregnancy or depression throughout pregnancy in any of the four models.

### Regional analysis

In regional analyses we identified 39 differentially methylated regions across all four depression exposures using the fully adjusted model (Sidak-corrected *p* < 0.05). Seven DMRs were associated with depression at any time point during pregnancy, 7 associated with depression in mid-pregnancy, 23 were associated with depression in late pregnancy, and 2 DMRs were associated with depression throughout pregnancy (Table 2).

**Table 2:** Results of the regional analysis

Exposure group	Gene	Location	No. of CpGs	DMR	Median beta (range)	Sidak-corrected *p*-values
Any time point	*RASIP1*	*19q13.33*	*5*	*Chr19:49223814–49224166*	*0.02 (0.01; 0.02)*	*2.8e-05*
	*HFM1*	*1p22.2*	*6*	*Chr1:91852791–91853090*	*−0.01 (−0.02; 0.0)*	*6.8e-04*
	*NNAT*	20q11.23	23	Chr20:36148604–36149082	−0.01 (−0.02; 0.00)	7.1e-04
	*HCG4P6*	*6p22.1*	*8*	*Chr6:29893926–29894163*	*0.04 (0.0; 0.07)*	*2.1e-03*
	*GNAS*	*20q13.32*	*9*	*3Chr20:57427556–57427831*	*0.01 (0.0; 0.02)*	*8.0e-03*
	*CARTPT*	*5q13.2*	*6*	*Chr5:71146767–71146877*	*−0.02 (−0.02; −0.00)*	*2.8e-02*
	*ARL5C*	17q12	3	Chr17:37322028–37322432	0.02 (0.01; 0.04)	5.3e-04
Mid-pregnancy depression	*HOXA5*	7p15.2	42	Chr7:27183133–27184522	−0.02 (−0.06; 0.00)	8.4e-10
	*LYNX1*	8q24.3	7	Chr8:143859669–143859991	0.03 (0.02; 0.06)	1.2e-05
	*SULF1*	8q13.2	8	Chr8:70378380–70378995	0.01 (0.00; 0.02)	8.0e-05
	*C6orf227*	6p21.31	8	Chr6:33561099–33561450	0.01 (0.00; 0.03)	1.5e-04
	*RGS1*	1q31.2	2	Chr1:192544716–192544903	0.04 (0.01; 0.07)	3.2e-03
	*BCL11B*	14q32.2	3	Chr14:99655593–99655748	−0.04 (−0.06; −0.03)	9.2e-03
	*SNED1*	2q37.3	2	Chr2:242031617–242031695	0.01 (−0.00; 0.02)	3.0e-02
Late depression	*CARTPT*	*5q13.2*	*6*	*Chr5:71146767–71146877*	*−0.03 (−0.04; −0.02)*	*6.6e-08*
	*HFM1*	*1p22.2*	*6*	*Chr1:91852791–91853090*	*−0.02 (−0.03; −0.00)*	*1.7e-06*
	*HCG4P6*	*6p22.1*	*16*	*Chr6:29893926–29894342*	*0.02 (0.00; 0.01)*	*1.8e-05*
	*PLEKHG1*	6q25.1	3	Chr6:151125848–151125886	0.03 (0.03; 0.03)	3.0e-05
	*FAM20C*	7p22.3	5	Chr7:213469–213849	−0.02 (−0.04; 0.00)	1.0e-04
	*DDO*	6q21	4	Chr6:110720918–110721350	0.05 (0.03; 0.06)	1.3e-04
	*RASIP1*	*19q13.33*	*5*	*Chr19:49223814–49224166*	*0.02 (0.01; 0.03)*	*1.7e-04*
	*HLA-DRB6*	6p21.32	14	Chr6:32551749–32552247	−0.05 (−0.10; −0.00)	2.5e-04
	*JA668105*	2q21.2	3	Chr2:133025652–133025918	−0.01 (−0.03; 0.03)	5.3e-04
	*TRNA_Ala*	6p22.1	17	Chr6:28583971–28584289	−0.01 (−0.03; −0.00)	6.1e-04
	*KLHL30*	2q317.3	6	Chr2:239047029–239047337	0.02 (0.01; 0.04)	1.1e-03
	*MUC4*	3q29	7	Chr3:195489708–195490170	0.05 (0.02; 0.07)	1.1e-03
	*JPH3*	16q24.2	2	Chr16:87682036–87682143	−0.08 (−0.01; −0.05)	3.5e-03
	*PSPH*	7p11.2	3	Chr7:56242181–56242555	0.04 (0.03; 0.04)	5.5e-03
	*AK054726*	1q42.2	5	Chr1:234367145–234367587	−0.03 (−0.04; −0.03)	6.4e-03
	*LMO7*	13q22.2	4	Chr13:76334583–76334867	0.02 (0.02; 0.02)	6.7e-03
	*ESRRG*	1q41	3	Chr1:217306590–217306764	−0.04 (−0.04; −0.03)	9.0e-03
	*SDK1*	7p22.2	3	Chr7:4244250–4244644	−0.06 (−0.01; −0.05)	1.0e-02
	*SLCO5A1*	8q13.3	4	Chr8:70602451–70602610	−0.01 (−0.03; 0.00)	2.0e-02
	*IFITM4P*	6p22.1	4	Chr6:29723301–29723408	−0.04 (−0.06; −0.04)	2.9e-02
	*DLG2*	11q14.1	3	Chr11:85195094–85195206	−0.01 (−0.02; −0.01)	3.1e-02
	*BIN1*	2q14.3	2	Chr2:127811805–127811854	−0.03 (−0.04; −0.03)	3.2e-02
	*DLX1*	2q31.1	2	Chr2:172948725–172948826	0.01 (0.01; 0.01)	4.8e-02
All	*GNA* *S*	*20q13.32*	*17*	*Chr20:57427472–57428033*	*0.02 (0.00; 0.03)*	*4.1e-09*
	*TRPM4*	19q13.33	4	Chr19:49713645–49714045	−0.03 (−0.05; −0.02)	1.1e-03

The italics boxes indicate DMRs that are common to more than one exposure

Three DMRs (mapping to *HFMI, CARTPT*, and *RASIP1K*) were common to two exposures: depression at any time point during pregnancy and depression in late pregnancy only. These two analyses also identified DMRs in close proximity to each other, but not identical. These DMRs both mapped to the *HCG4P6* gene.

The regional analyses in women with depression at any time point during pregnancy and depression throughout pregnancy did not share mutual DMRs. They did however identify neighbouring DMRs that both mapped to the *GNAS* gene.

No mutual DMRs were identified in the analyses of the cord blood from offspring having been exposed to depression in mid-pregnancy, late, and throughout pregnancy, respectively.

### Functional gene enrichment analysis (GO and KEGG analyses)

Among the DMRs specific to depression throughout pregnancy we found 1 KEGG pathway (related to insulin secretion) but no enriched biological process GO terms (FDR < 0.05).

### Replication

Our look up of the results from the single site analysis and the regional analysis in ALSPAC did not replicate in the Generation R sample.

Of the two CpG sites (cg08667740 and cg22868225) that were specific to mid-pregnancy maternal depression in ALSPAC, one was associated with depression in Generation R with the same direction of effect. The other CpG was associated with depression with the opposite direction of effect. None of them reached statistical significance (Table 3).

**Table 3:** Table of replication in Generation R of the significant single CpG sites found in ALSPAC

Probe ID	ALSPAC mid-pregnancy depression			Generation R Study		
	Beta	SE	*p*-value	Beta	SE	*p*-value
cg08667740	−0.025	0.005	3.90E-08	0.003	0.002	0.186
cg22868225	−0.005	0.001	5.89E-08	−0.001	0.001	0.672

In the regional analysis we found no mutual DMRs in the two datasets. Out of the 68 CpGs within the seven DMRs specific to depression in mid-pregnancy in the ALSPAC regional analysis, 49 (72%) showed the same direction of association in the Generation R study, but none survived correction for multiple testing (Tables S2, S3).

### Methylation quantative trait loci analysis

Of the two CpGs identified in our single site analysis, cg22868225 was associated with two trans-SNPs located on chromosome 10 (rs17569335, *β* = 0.267, *p* = 7.8e-08 and rs2768469, *β* = 0.276, *p* = 3.41e-08), and cg08667740 was associated with one trans-acting SNP (rs13190610, *β* = −1.352, *p* = 2.04e-08) located on chromosome 5.

Neither CpG was associated with any *cis*-acting SNPs, suggesting that the observed methylation differences between offspring of depressed and unaffected mothers is not being driven by the underlying genetic architecture.

## Discussion

Maternal depression (occurring at any time point during pregnancy) was associated with offspring cord blood DNA methylation at seven DMRs, but no individual CpG sites in our main analysis in ALSPAC.

Depression in mid-pregnancy, late pregnancy, and at both time points (throughout pregnancy) was associated with 7, 23, and 2 DMRs, respectively. Depression in mid-pregnancy was the only exposure definition associated with any individual CpG sites with genome-wide significance. However, findings for the two identified CpGs (cg08667740 and cg22868225) did not replicate in Generation R. Although we found that 72% CpG sites within DMRs identified in ALSPAC showed the same direction of association between antenatal maternal depression and offspring DNA methylation in Generation R, none of our findings replicated with statistical significance.

Although none of the ALSPAC DMRs replicated in the Generation R study with statistical significance, the direction of association was the same at some regions that have previously been associated with neurodevelopmental and psychiatric outcomes. Out of the 49 CpG sites that had the same direction of association in both ALSPAC and the Generation R study 35 were hypomethylated in both cohorts and located in the promoter region of the *HOXA5* gene. This gene has been associated with depression in early pregnancy and this gene family is known to have important roles in embryonic development^42^ and has been seen to be downregulated in the prefrontal cortex of depressed suicide subjects^43^.

Another seven CpG sites that were all hypermethylated in both ALSPAC and the Generation R study mapped to the promoter region of the *LYNX1* gene. This gene was previously found to be hypermethylated in the hippocampus from patients with major depressive disorders^44^. In this study they conducted both a genome-wide association study using the 450K array and investigated the *LYNX1* gene using bisulfite pyrosequencing. Both methods yielded the same result.

In the cord blood of the children exposed to depression at any time point in pregnancy we found a DMR spanning more than 20 CpG sites mapping to the promotor region of the *NNAT* gene which is an imprinted gene that may be involved in brain development and in forming and maintaining the structure of the nervous system^45^.

The *NNAT* gene has been investigated previously in relation to maternal depression during pregnancy^46,47^. These studies investigated imprinted genes but none of them found any association between depression during pregnancy and changes in DNA methylation in cord blood of newborn infants in this gene.

In candidate gene studies, Devlin et al.^13^ and Oberlander et al.^14^ found associations between maternal depressed mood during pregnancy and variation in cord blood DNA methylation at the *SLC6A4* and *NR3C1* genes, respectively. These findings were not replicated in our study.

There has been one previous EWAS of maternal depressed mood or anxiety during pregnancy and cord blood methylation in just 13 children^48^. Of the 10 CpG sites differentially methylated with FDR < 0.05 in this study, 5 CpG sites were associated with depression in the same direction in our study, but the smallest FDR-corrected *p*-value for these associations was 0.999 (Table S4).

### Strengths and limitations

The ALSPAC study is a large birth cohort with very comprehensive, prospectively collected data on a population level reducing the possibility of recall bias or reverse causality.

Another strength to this study is the use of the EPDS, which is a self-reported screening tool for depression that correlates with clinical depression with a high sensitivity at the chosen cut off of 12 points^19^. However, it should be noted that this is a screening tool and therefore the prevalence of depression (20%) in our study was higher than estimates for clinical diagnoses (~13%)^1^. It is interesting that we find at least some association between depression and offspring methylation in our study, given that women in our study are likely to have with milder forms of depression. In future studies it would be interesting to investigate the association between exposure to clinically verified depression and changes in DNA methylation in cord blood from the exposed infants.

A limitation to this study is the coverage of the 450K BeadChip. It covers less than 2% of the CpG sites in the human epigenome. This limited coverage could result in an underestimation of the true methylation differences in epigenomic areas not covered by this array, including methylation variable loci that have previously been implicated in depression.

Even though, to our knowledge, this study is the largest on depression during pregnancy and cord blood methylation, inability to detect true associations in our discovery (ALSPAC) and replication (The Generation R Study) cohorts due to lack of statistical power is entirely possible. Furthermore, the Generation R Study used a different measure of depression to the ALSPAC cohort. The correlation between the two different mood scores used by ALSPAC (EPDS) and The Generation R Study (BSI) has been studied sparsely^49^ and the relatively low may correlation contribute to the lack of replication. Using two different instruments may however be considered a strength in this study. The convergent direction of associations between the cohorts point towards an effect of depression rather than an instrument specific effect.

The methylation data used in this study are derived from cord blood, and it could be argued that it would be more appropriate to study the association of maternal depression and DNA methylation in a more biologically relevant tissue, for example, infant brain tissue. However, aside from the obvious ethical and feasibility issues, there is increasing evidence to suggest that peripheral tissues may have some utility in epigenetic epidemiological studies of brain disorders^50,51^.

The composition of cells in umbilical cord blood may confound results if the composition is related to exposure or outcome. In this study, it is plausible that maternal depression, or exposures related to depression, such as smoking, could influence cell composition in cord blood. In order to limit the effect of this we adjusted for cord blood cell composition using the Andrews and Bakulski cord blood reference panel. We also investigated the association between exposure and estimated cell proportion. Maternal depression was not associated with the estimated cell proportions (Table S5).

We aknowledge, that it could be a limitation to this study, that we have not confirmed the significant CpG sites by more quantitative analyses, e.g. pyrosequencing. The 450K array is however well validated, and yields values consistent with pyrosequencing^52^.

We have chosen the included covariates using the theory of DAGs and have therefore not included covariates often included in previous studies such as, e.g. child-sex and gestational age that are not true confounders with direct effect on both exposure and outcome. Conditioning on such covariates could confound the results and either over- or underestimate the true association depending on the source of bias^53,54^.

The possibility of residual confounding cannot be ruled out but the sample size was not sufficient for the addition of more variables to the analysis.

However, despite this, to our knowledge, being the largest study of maternal depression and cord blood methylation to date, the sample size was not sufficient to allow for more stratified analyses or the addition of more variables to the multivariable analyses.

More smokers were found among depressed women compared to controls. Thus, residual confounding by smoking may exist. However, we found no overlap between the top CpG sites found in our analyses of depressed women vs. controls and the smoking-associated CpG sites from a previous study^55^. This suggests that smoking may not be a strong confounder in our study.

Finally, the effect sizes in this study are rather small. The biological significance of this is unclear, and could not be explored further due to the lack of data such as downstream gene expression. Other environmental epigenetic studies do show that often only small-magnitude effects results from these exposures^56^, and future studies should evaluate the significance of these.

## Conclusion

We identified some single and regional DNA methylation differences in umbilical cord blood of newborns exposed to maternal depression during pregnancy, compared to newborns that were unexposed but these results did not replicate in an independent cohort. Further studies with larger sample sizes and better epigenomic coverage investigating more tissue types are needed to further investigate whether epigenetics could be part of a biological pathway linking maternal depression during pregnancy to childhood outcomes.

## Electronic supplementary material

Table S1. Characteristics of the included women in the replication in Generation R Study

Table S2. Table of replication results for CpGs in DMRs identified in the ALSPAC study of anytime depression

Table S3. Table of replication results for CpGs in DMRs identified in the ALSPAC study of depression in mid-pregnancy

Table S4.CPG-sites found associated with maternal depression in the study by Non et al

Table S5: Cell type proportion by depression level
