# Supplementary material for: Maternal depression during pregnancy and cord blood DNA methylation: findings from the Avon Longitudinal Study of Parents and Children
Source: Transl Psychiatry. 2018 Nov 7;8:244. doi: 10.1038/s41398-018-0286-4 (PMC6221892; doi:10.1038/s41398-018-0286-4)
Supplement: Supplementary file 1 — Table S1. Characteristics of the included women in the replication in Generation R Study [file 41398_2018_286_MOESM1_ESM.docx]

**Table S1. Characteristics of the included women in the replication in Generation R Study.**

| N=1038 | **Depression at 20 weeks of pregnancy**  N=33  (BSI depression score  > 0.80) | **No depression at 20 weeks of pregnancy**  N=1005  (BSI depression score  ≤ 0.80) |
| --- | --- | --- |
| **Age (years) (mean(95%CI))** | 31.4(18.7;39.1) | 32.4 (16.8;46.9) |
| **BMI (mean (95%CI))** | 23.7(18.0;38.0) | 23.2(17.3; 43.3) |
| **Parity (n)** |  |  |
| No children | 20(61%) | 611(61%) |
| ≥1 child | 13 (39%) | 394(39%) |
| **Smoking in pregnancy (n)** |  |  |
| No smoking | 20(61%) | 786(78%) |
| Any smoking | 13(39%) | 219(22%) |
| **Education Level** |  |  |
| Below A -levels | 8(24%) | 98(10%) |
| A-levels and above | 25(76%) | 907(90%) |
