# Supplementary material for: Maternal depression during pregnancy and cord blood DNA methylation: findings from the Avon Longitudinal Study of Parents and Children
Source: Transl Psychiatry. 2018 Nov 7;8:244. doi: 10.1038/s41398-018-0286-4 (PMC6221892; doi:10.1038/s41398-018-0286-4)
Supplement: Supplementary file 2 — Table S2. Table of replication results for CpGs in DMRs identified in the ALSPAC study of anytime depression [file 41398_2018_286_MOESM2_ESM.docx]

**Table S2. Table of replication results for CpGs in DMRs identified in the ALSPAC study of anytime depression. 27 out of 58 CpG-sites showed the same direction of effect in both ALSPAC and Generation R (shaded rows).**

|  | | | **ALSPAC- anytime** | | **The Generation R Study** | |
| --- | --- | --- | --- | --- | --- | --- |
| **DMR** | **Gene** | **Probe ID** | **Beta-value** | **P-value** | **Beta-value** | **P-value** |
| Chr17:37322028-37322432 | ARL5C | cg00900933 | 0.0102651 | 5.48E-03 | -0.0087824 | 0.164713 |
| Chr17:37322028-37322432 | ARL5C | cg09173348 | 0.0236357 | 0.006197 | -0.0181368 | 0.061517 |
| Chr17:37322028-37322432 | ARL5C | cg07330481 | 0.0365987 | 0.0001904 | -0.0071051 | 0.580925 |
| Chr19:49223814-49224166 | RASIP1 | cg13974464 | 0.019631 | 0.0072616 | -0.0018607 | 0.891828 |
| Chr19:49223814-49224166 | RASIP1 | cg10495207 | 0.0119449 | 0.0061981 | -0.0037935 | 0.593472 |
| Chr19:49223814-49224166 | RASIP1 | cg13428516 | 0.0133987 | 0.0387748 | -0.000952 | 0.922773 |
| Chr19:49223814-49224166 | RASIP1 | cg02045294 | 0.0151326 | 0.0005309 | 0.0012116 | 0.834285 |
| Chr1:91852791-91853090 | HFM1 | cg08105529 | -0.0062738 | 0.0017265 | -0.0031592 | 0.452375 |
| Chr1:91852791-91853090 | HFM1 | cg19082970 | -0.0102565 | 0.0087172 | -0.0099199 | 0.245079 |
| Chr1:91852791-91853090 | HFM1 | cg00070162 | 0.0049249 | 0.2809233 | 0.0027994 | 0.588888 |
| Chr1:91852791-91853090 | HFM1 | cg00162191 | -0.0162695 | 0.005415 | 0.0007675 | 0.943743 |
| Chr1:91852791-91853090 | HFM1 | cg21144650 | -0.0120704 | 0.0082979 | -0.0032914 | 0.65322 |
| Chr1:91852791-91853090 | HFM1 | cg20256120 | -0.0067019 | 0.0416671 | -0.0048194 | 0.54414 |
| Chr20:36148604-36149082 | NNAT | cg14765818 | -0.0091727 | 0.0132367 | 0.0134903 | 0.024362 |
| Chr20:36148604-36149082 | NNAT | cg12862537 | -0.0097332 | 0.2857783 | 0.0167947 | 0.048248 |
| Chr20:36148604-36149082 | NNAT | cg20783699 | -0.0166318 | 0.075454 | 0.0119533 | 0.155231 |
| Chr20:36148604-36149082 | NNAT | cg04489586 | -0.0082648 | 0.033737 | 0.0153949 | 0.010327 |
| Chr20:36148604-36149082 | NNAT | cg15473473 | -0.0030642 | 0.7622163 | 0.0106648 | 0.208848 |
| Chr20:36148604-36149082 | NNAT | cg22298088 | -0.0167285 | 0.1121661 | 0.0061156 | 0.496495 |
| Chr20:36148604-36149082 | NNAT | cg17643025 | -0.0110395 | 0.2235818 | 0.0123006 | 0.078193 |
| Chr20:36148604-36149082 | NNAT | cg07156273 | -0.0075029 | 0.4602847 | 0.005551 | 0.539922 |
| Chr20:36148604-36149082 | NNAT | cg25712981 | -0.0220476 | 0.0330083 | 0.0154595 | 0.072649 |
| Chr20:36148604-36149082 | NNAT | cg22551578 | -0.010206 | 0.2868252 | 0.0213957 | 0.090391 |
| Chr20:36148604-36149082 | NNAT | cg22943498 | 0.001092 | 0.9348119 | 0.0221415 | 0.09668 |
| Chr20:36148604-36149082 | NNAT | cg23605670 | -0.0030212 | 0.7008658 | 0.0094391 | 0.243952 |
| Chr20:36148604-36149082 | NNAT | cg01466133 | -0.0163443 | 0.0851849 | 0.015019 | 0.101591 |
| Chr20:36148604-36149082 | NNAT | cg26083330 | -0.014313 | 0.0216567 | 0.0125052 | 0.138639 |
| Chr20:36148604-36149082 | NNAT | cg24338351 | -0.0122145 | 0.009066 | 0.0093732 | 0.148241 |
| Chr20:36148604-36149082 | NNAT | cg10981598 | -0.0097545 | 0.256545 | 0.0180528 | 0.032244 |
| Chr20:36148604-36149082 | NNAT | cg08402058 | -0.0122573 | 0.0010048 | 0.0122845 | 0.030964 |
| Chr20:36148604-36149082 | NNAT | cg23757721 | -0.0151645 | 0.0323984 | 0.0218747 | 0.009728 |
| Chr20:36148604-36149082 | NNAT | cg13790727 | -0.0121714 | 0.0109269 | 0.0143587 | 0.027127 |
| Chr20:36148604-36149082 | NNAT | cg14469070 | -0.019065 | 0.0176956 | 0.0080147 | 0.217441 |
| Chr20:36148604-36149082 | NNAT | cg24762053 | -0.0116941 | 0.0800915 | 0.0063993 | 0.236693 |
| Chr20:36148604-36149082 | NNAT | cg24675557 | -0.0176572 | 0.1015075 | 0.0110239 | 0.297815 |
| Chr20:57427556-57427831 | GNAS | cg19589727 | 0.008841 | 0.0087918 | 0.0015288 | 0.810857 |
| Chr20:57427556-57427831 | GNAS | cg01817393 | 0.010715 | 0.1538051 | 0.0031657 | 0.840966 |
| Chr20:57427556-57427831 | GNAS | cg02890368 | 0.0056051 | 0.1325746 | 0.0177288 | 0.310782 |
| Chr20:57427556-57427831 | GNAS | cg27661264 | 0.0069603 | 0.0455463 | 0.0076782 | 0.245769 |
| Chr20:57427556-57427831 | GNAS | cg10302550 | 0.0109833 | 0.043818 | 0.0026393 | 0.712489 |
| Chr20:57427556-57427831 | GNAS | cg04257105 | 0.0116186 | 0.0262135 | 0.0045497 | 0.574826 |
| Chr20:57427556-57427831 | GNAS | cg14564778 | -0.0016314 | 0.8218253 | 0.0120409 | 0.239375 |
| Chr20:57427556-57427831 | GNAS | cg17414107 | 0.0155018 | 0.0046679 | 0.004776 | 0.559218 |
| Chr20:57427556-57427831 | GNAS | cg20528838 | 0.0132572 | 0.0021026 | 0.0029545 | 0.696627 |
| Chr5:71146767-71146877 | CARTPT | cg11806367 | -0.0040079 | 0.5985494 | -0.0218995 | 0.191089 |
| Chr5:71146767-71146877 | CARTPT | cg07761367 | -0.0078138 | 0.0054221 | -0.004612 | 0.451698 |
| Chr5:71146767-71146877 | CARTPT | cg05696584 | -0.0198276 | 0.0092353 | -0.0104551 | 0.318411 |
| Chr5:71146767-71146877 | CARTPT | cg00597112 | -0.0178574 | 0.0097802 | -0.0049715 | 0.635408 |
| Chr5:71146767-71146877 | CARTPT | cg07820786 | -0.0219594 | 0.0037728 | -0.008086 | 0.505791 |
| Chr5:71146767-71146877 | CARTPT | cg08285730 | -0.0116735 | 0.0379497 | -0.007684 | 0.397221 |
| Chr6:29893926-29894163 | BC035647 | cg27165617 | 0.000409 | 0.8867512 | 0.0051944 | 0.471544 |
| Chr6:29893926-29894163 | BC035647 | cg15070894 | 0.0595645 | 0.0017709 | 0.0077251 | 0.839783 |
| Chr6:29893926-29894163 | BC035647 | cg10595298 | 0.0206258 | 0.0011035 | 0.0048928 | 0.687025 |
| Chr6:29893926-29894163 | BC035647 | cg24751894 | 0.055705 | 0.0072142 | -0.0066805 | 0.868521 |
| Chr6:29893926-29894163 | BC035647 | cg25644740 | 0.0704189 | 0.0047583 | 0.0182644 | 0.716011 |
| Chr6:29893926-29894163 | BC035647 | cg04227238 | 0.0077153 | 0.4025174 | 0.0164768 | 0.405888 |
| Chr6:29893926-29894163 | BC035647 | cg16302021 | 0.0075226 | 0.1012641 | -0.0038998 | 0.649698 |
| Chr6:29893926-29894163 | BC035647 | cg18786623 | 0.0730148 | 0.0014281 | 0.007411 | 0.860273 |
