# Supplementary material for: Maternal depression during pregnancy and cord blood DNA methylation: findings from the Avon Longitudinal Study of Parents and Children
Source: Transl Psychiatry. 2018 Nov 7;8:244. doi: 10.1038/s41398-018-0286-4 (PMC6221892; doi:10.1038/s41398-018-0286-4)
Supplement: Supplementary file 3 — Table S3. Table of replication results for CpGs in DMRs identified in the ALSPAC study of depression in mid-pregnancy [file 41398_2018_286_MOESM3_ESM.docx]

**Table S3. Table of replication results for CpGs in DMRs identified in the ALSPAC study of depression in mid-pregnancy. 49 out of 68 CpG-sites showed the same direction of effect in both ALSPAC and Generation R (shaded rows).**

|  | | | **ALSPAC- mid-pregnancy** | | **The Generation R Study** | |
| --- | --- | --- | --- | --- | --- | --- |
| **DMR** | **Gene** | **Probe ID** | **Beta-value** | **P-value** | **Beta-value** | **P-value** |
| Chr14:99655593-99655748 | BCL11B | cg04712122 | -0.0364929 | 0.0174914 | 0.0008737 | 0.944436 |
| Chr14:99655593-99655748 | BCL11B | cg07015803 | -0.0319321 | 0.0194796 | -0.0032043 | 0.613984 |
| Chr16:88711583-88711584 | CYBA | cg08667740 | -0.0251167 | 3.90E-08 | 0.0032954 | 0.186401 |
| Chr1:192544716-192544903 | RGS1 | cg02586212 | 0.0692113 | 0.0000159 | -0.00072 | 0.971104 |
| Chr1:192544716-192544903 | RGS1 | cg10861751 | 0.0109117 | 0.0061035 | -0.0038142 | 0.402565 |
| Chr2:242031617-242031695 | SNED1 | cg14454796 | -0.0030229 | 0.0026258 | -0.0003033 | 0.797025 |
| Chr2:242031617-242031695 | SNED1 | cg24407092 | 0.016737 | 0.0002264 | -0.003496 | 0.150867 |
| Chr6:33561099-33561450 | LINC00336 | cg08301503 | 0.0129382 | 0.0074915 | -0.0011148 | 0.87743 |
| Chr6:33561099-33561450 | LINC00336 | cg00536532 | 0.0129884 | 0.0923981 | -0.0060569 | 0.266014 |
| Chr6:33561099-33561450 | LINC00336 | cg06289138 | 0.014705 | 0.0125535 | 0.0032529 | 0.73971 |
| Chr6:33561099-33561450 | LINC00336 | cg19869469 | 0.0100255 | 0.01926 | -0.0058969 | 0.287157 |
| Chr6:33561099-33561450 | LINC00336 | cg04329454 | 0.0261803 | 0.0159098 | 0.0146238 | 0.370071 |
| Chr6:33561099-33561450 | LINC00336 | cg01392313 | 0.0175402 | 0.0004761 | 0.002314 | 0.808601 |
| Chr6:33561099-33561450 | LINC00336 | cg05602975 | 0.003065 | 0.5290621 | 0.0022678 | 0.709087 |
| Chr6:33561099-33561450 | LINC00336 | cg07873320 | 0.0123374 | 0.0165951 | -0.0001766 | 0.979846 |
| Chr7:27183133-27184522 | HOXA6 | cg02106682 | -0.0132202 | 0.0970011 | -0.0052357 | 0.461464 |
| Chr7:27183133-27184522 | HOXA5 | cg02005600 | -0.0384048 | 0.0659985 | -0.0170237 | 0.211374 |
| Chr7:27183133-27184522 | HOXA5 | cg12128839 | -0.0539383 | 0.0421881 | -0.0162249 | 0.392581 |
| Chr7:27183133-27184522 | HOXA5 | cg19196335 | -0.0172398 | 0.0221601 | -0.0003082 | 0.929032 |
| Chr7:27183133-27184522 | HOXA6 | cg26023912 | -0.0172973 | 0.1183956 | -0.016499 | 0.107783 |
| Chr7:27183133-27184522 | HOXA5 | cg25866143 | -0.0372651 | 0.0302221 | -0.0173356 | 0.123131 |
| Chr7:27183133-27184522 | HOXA5 | cg16997642 | -0.0123157 | 0.154984 | -0.0032482 | 0.537607 |
| Chr7:27183133-27184522 | HOXA5 | cg14013695 | -0.0141063 | 0.2602574 | -0.0119012 | 0.193357 |
| Chr7:27183133-27184522 | HOXA5 | cg19759481 | -0.0352676 | 0.1571182 | -0.0134145 | 0.432847 |
| Chr7:27183133-27184522 | HOXA5 | cg14658493 | -0.0335535 | 0.0014987 | -0.0122522 | 0.199932 |
| Chr7:27183133-27184522 | HOXA5 | cg09549073 | -0.0403338 | 0.0278744 | -0.0241498 | 0.077261 |
| Chr7:27183133-27184522 | HOXA5 | cg20817131 | -0.01268 | 0.2903801 | -0.0162043 | 0.074676 |
| Chr7:27183133-27184522 | HOXA5 | cg25506432 | 0.001246 | 0.9289662 | -0.0073194 | 0.419218 |
| Chr7:27183133-27184522 | HOXA6 | cg03368099 | -0.0256607 | 0.0582779 | -0.0172908 | 0.101798 |
| Chr7:27183133-27184522 | HOXA5 | cg23204968 | -0.0092167 | 0.4045631 | -0.0012133 | 0.903146 |
| Chr7:27183133-27184522 | HOXA5 | cg09880291 | -0.0050128 | 0.5411065 | -0.007655 | 0.096909 |
| Chr7:27183133-27184522 | HOXA5 | cg23454797 | -0.0131106 | 0.2966705 | -0.0070445 | 0.373233 |
| Chr7:27183133-27184522 | HOXA5 | cg25390165 | -0.0329841 | 0.0055791 | -0.0119387 | 0.146606 |
| Chr7:27183133-27184522 | HOXA5 | cg02646423 | -0.0095121 | 0.5548779 | -0.0114136 | 0.28764 |
| Chr7:27183133-27184522 | HOXA6 | cg01323381 | -0.0257742 | 0.0735733 | -0.0119356 | 0.197926 |
| Chr7:27183133-27184522 | HOXA6 | cg17432857 | -0.0211634 | 0.2287325 | -0.0209415 | 0.07368 |
| Chr7:27183133-27184522 | HOXA5 | cg14014955 | -0.016652 | 0.2484903 | -0.0165652 | 0.153522 |
| Chr7:27183133-27184522 | HOXA5 | cg04863892 | -0.0454067 | 0.0679636 | -0.0153598 | 0.327415 |
| Chr7:27183133-27184522 | HOXA5 | cg08070327 | -0.0148349 | 0.0059971 | 0.0031107 | 0.623526 |
| Chr7:27183133-27184522 | HOXA5 | cg05835726 | -0.0415161 | 0.0221004 | -0.0195555 | 0.123463 |
| Chr7:27183133-27184522 | HOXA5 | cg23936031 | -0.0503578 | 0.0109837 | -0.0297524 | 0.046402 |
| Chr7:27183133-27184522 | HOXA5 | cg20517050 | -0.0290058 | 0.0529731 | -0.0190144 | 0.204507 |
| Chr7:27183133-27184522 | HOXA6 | cg07049592 | -0.0098547 | 0.2471757 | -0.0013722 | 0.838868 |
| Chr7:27183133-27184522 | HOXA6 | cg00969405 | -0.027084 | 0.0922338 | -0.0197186 | 0.077657 |
| Chr7:27183133-27184522 | HOXA6 | cg05774699 | -0.0214051 | 0.0379671 | -0.0098928 | 0.144061 |
| Chr7:27183133-27184522 | HOXA5 | cg25307665 | -0.0405172 | 0.0942264 | -0.0266746 | 0.156128 |
| Chr7:27183133-27184522 | HOXA5 | cg03207666 | -0.0109281 | 0.1071359 | -0.0020173 | 0.646553 |
| Chr7:27183133-27184522 | HOXA5 | cg24389585 | -0.0010936 | 0.8933715 | 0.0001163 | 0.972976 |
| Chr7:27183133-27184522 | HOXA5 | cg14058329 | -0.0087581 | 0.3257179 | -0.0123164 | 0.07884 |
| Chr7:27183133-27184522 | HOXA6 | cg19643053 | -0.0068496 | 0.3362741 | -0.0016721 | 0.704447 |
| Chr7:27183133-27184522 | HOXA5 | cg12015737 | -0.0287695 | 0.0175652 | 0.004627 | 0.50403 |
| Chr7:27183133-27184522 | HOXA5 | cg17569124 | -0.0500209 | 0.0140013 | -0.0101292 | 0.520153 |
| Chr7:27183133-27184522 | HOXA5 | cg02916332 | -0.0365149 | 0.0096994 | -0.0198837 | 0.110267 |
| Chr7:27183133-27184522 | HOXA6 | cg14882265 | -0.0203423 | 0.0908555 | -0.0088049 | 0.411179 |
| Chr8:143859669-143859991 | LYNX1 | cg10555383 | 0.0341075 | 0.0015052 | 0.0176991 | 0.233133 |
| Chr8:143859669-143859991 | LYNX1 | cg23180489 | 0.0425357 | 0.0066017 | 0.0175788 | 0.140694 |
| Chr8:143859669-143859991 | LYNX1 | cg00399059 | 0.0553533 | 0.0011298 | 0.0185917 | 0.149639 |
| Chr8:143859669-143859991 | LYNX1 | cg19021188 | 0.0230286 | 0.1495806 | 0.016822 | 0.154549 |
| Chr8:143859669-143859991 | LYNX1 | cg08090164 | 0.0257993 | 0.0224904 | 0.0102751 | 0.19191 |
| Chr8:143859669-143859991 | LYNX1 | cg25483741 | 0.0298773 | 0.0274119 | 0.0094391 | 0.35969 |
| Chr8:143859669-143859991 | LYNX1 | cg22861548 | 0.0454159 | 0.0122287 | 0.0136041 | 0.338177 |
| Chr8:70378380-70378995 | SULF1 | cg07051728 | 0.009586 | 0.0740512 | -0.0003685 | 0.94972 |
| Chr8:70378380-70378995 | SULF1 | cg04845579 | 0.0215046 | 0.0360943 | -0.005413 | 0.677332 |
| Chr8:70378380-70378995 | SULF1 | cg05806180 | 0.009308 | 0.0011204 | -0.0004073 | 0.952755 |
| Chr8:70378380-70378995 | SULF1 | cg07073960 | 0.0118473 | 0.0113325 | -0.0068937 | 0.445126 |
| Chr8:70378380-70378995 | SULF1 | cg12181083 | 0.0141454 | 0.0060869 | -0.0018086 | 0.86286 |
| Chr8:70378380-70378995 | SULF1 | cg00613562 | 0.0017067 | 0.7141458 | -0.0076414 | 0.414156 |
| Chr8:70378380-70378995 | SULF1 | cg15351186 | 0.008416 | 0.0810748 | 0.0085167 | 0.288255 |
