# Supplementary material for: Maternal depression during pregnancy and cord blood DNA methylation: findings from the Avon Longitudinal Study of Parents and Children
Source: Transl Psychiatry. 2018 Nov 7;8:244. doi: 10.1038/s41398-018-0286-4 (PMC6221892; doi:10.1038/s41398-018-0286-4)
Supplement: Supplementary file 4 — Table S4.CPG-sites found associated with maternal depression in the study by Non et al [file 41398_2018_286_MOESM4_ESM.docx]

Table S4.CPG-sites found associated with maternal depression in the study by Non et al. compared to the same CpG-sites in the ALSPAC study of anytime depression in pregnancy.

|  | Non et al. 2012 | |  | ALSPAC-anytime | |
| --- | --- | --- | --- | --- | --- |
| Probe ID | **Beta-value** | **FDR corrected P-value** |  | **Beta-value** | **FDR corrected P-value** |
| cg17913386 | -0.087 | 0.046 |  | -0.00259 | 0.999 |
| cg11846236 | -0.080 | 0.046 |  | 0.00084 | 0.999 |
| cg04556542 | -0.015 | 0.046 |  | -0.00027 | 0.999 |
| cg25109393 | -0.011 | 0.046 |  | 0.00019 | 0.999 |
| cg21675030 | -0.010 | 0.046 |  | 0.00009 | 0.999 |
| cg24141135 | -0.009 | 0.046 |  | 0.00012 | 0.999 |
| cg27180315 | -0.008 | 0.046 |  | 0.00024 | 0.999 |
| cg01070078 | 0.009 | 0.046 |  | 0.00018 | 0.999 |
| cg10283969 | 0.032 | 0.046 |  | 0.00267 | 0.999 |
| cg20242129 | 0.036 | 0.046 |  | 0.00115 | 0.999 |
