# Supplementary material for: Maternal depression during pregnancy and cord blood DNA methylation: findings from the Avon Longitudinal Study of Parents and Children
Source: Transl Psychiatry. 2018 Nov 7;8:244. doi: 10.1038/s41398-018-0286-4 (PMC6221892; doi:10.1038/s41398-018-0286-4)
Supplement: Supplementary file 5 — Table S5: Cell type proportion by depression level [file 41398_2018_286_MOESM5_ESM.docx]

**Table S5: Cell type proportion by depression level**

| **Cell type** | **Estimated cell proportion (Mean (95% CI))**  **EPDS < 12** | **Estimated cell proportion (Mean (95% CI))**  **EPDS≥12** | **P-value** |
| --- | --- | --- | --- |
| **B cells** | 0.17 (0.17-0.17) | 0.17 (0.16-0.18) | 0.74 |
| **CD4^+^T cells** | 0.18(0.17-0.18) | 0.17(0.15-0.19) | 0.66 |
| **CD8^+^T cells** | 0.09(0.09-0.09) | 0.09(0.08-0.1) | 0.89 |
| **Granulocytes** | 0.35(0.34-0.36) | 0.36(0.32-0.39) | 0.83 |
| **Natural killer cells** | 0.01(0.01-0.01) | 0.003(0.00-0.01) | 0.06 |
| **Nucleated red blood cells** | 0.20(0.19-0.21) | 0.20(0.17-0.24) | 0.76 |
| **Monocytes** | 0.01(0.01-0.01) | 0.01(0.01-0.01) | 0.23 |
